# Antimicrobial resistance profile of *Escherichia coli* isolated from poultry litter

**DOI:** 10.1016/j.psj.2022.102305

**Published:** 2022-11-09

**Authors:** M.J. Khong, A.M. Snyder, A.K. Magnaterra, M.M. Young, N.L. Barbieri, S.L. Weimer

**Affiliations:** ⁎ Department of Animal and Avian Sciences, University of Maryland, College Park, MD, 20742, USA; † Department of Population Health, University of Georgia College of Veterinary Medicine, Athens, GA, 30602, USA; ‡ Department of Poultry Science, University of Arkansas, Fayetteville, AR, 72701, USA

**Keywords:** *Escherichia coli*, APEC, poultry litter, antimicrobial resistance, disc diffusion

## Abstract

Antimicrobial resistance is a threat to animal and human health. As a commensal and zoonotic bacterium, *Escherichia coli* has the potential to be a pathogenic source of antimicrobial resistance. The purpose of this study aimed to investigate the antimicrobial resistance profile of *E. coli* isolated from litter collected from pens in a broiler chicken experiment. *E. coli* was isolated from litter samples (n = 68 isolates) of 16 pens housing broilers to d 53 of age. Resistance to 10 antimicrobials was observed by disc diffusion. The presence of 23 antimicrobial and heavy metal resistance genes, O serogroups, and avian pathogenic *E. coli* (APEC-like) minimal predictor genes were identified through PCR. *E. coli* isolates presented the greatest resistance to cephalothin (54.4%), tetracycline (27.9%), streptomycin (29.4%), ampicillin (20.6%), colistin (13.2%), sulphonamides (8.8%), and imipenem (1.5%). Multidrug resistance to at least 3 antimicrobials was observed in 22.1% of isolates. The identified O-types of the *E. coli* isolates were O15, O75, O78, and O91. There was a greater likelihood that the genes *groEL, aph(3)IA, silP, sull, aadA, qacEdelta1, iroN, ompTp,* and *hlyF* were present in isolates that exhibited ampicillin resistance (*P* ≤ 0.05). There was a greater likelihood that the *groEL* gene was present in isolates resistant to ampicillin, colistin, tetracycline, sulphonamides, or cephalothin (*P* ≤ 0.05). Further characterizing *E. coli* antimicrobial resistance is essential and aids in developing effective solutions, thereby furthering the One Health objective.

## INTRODUCTION

Antimicrobial resistance is one of the most important global health issues because it affects human and animal populations (World Health Organization, 2019). The U.S. Food and Drug Administration reported that 2.8 million people in the United States contracted an antimicrobial-resistant infection in 2019, leading to over 35,000 deaths (United States Food and Drug Administration, 2019). The One Health concept arises from the intersections of animals, humans, and the environment and aims to attain optimal public health by preventing and controlling zoonotic diseases (OHITF. One Health Initiative Task Force 2008; Bidaisee and Macpherson, 2014). Infections caused by antimicrobial-resistant bacteria can exacerbate existing complications in treatment and increase mortality in humans and animals (Munita and Arias, 2016).

Commonly found as a commensal (nonpathogenic) organism, *E. coli* can become pathogenic by acquiring virulence factors on plasmids or other mobile genetic elements via horizontal gene transfer, thus enabling certain strains of *E. coli* to cause intestinal or extraintestinal disease (Kaper et al., 2004; de Oliveira et al., 2020). A pathotype of concern in the poultry industry is avian pathogenic *E. coli* (**APEC**), which is the etiological agent of colibacillosis and can manifest as infections such as airsacculitis, polyserositis, and septicemia (Dho-Moulin and Fairbrother, 1999). Colibacillosis negatively impacts the health and welfare of poultry and incidence can be related to the quality of feed, water and litter, antimicrobial use and stewardship, and management practices (Nolan et al., 2013). Morbidity from *E. coli* infections can increase the use of antimicrobials for therapeutic treatment, resulting in significant economic losses for poultry producers. Mounting evidence suggests that APEC-contaminated poultry is a source of extraintestinal pathogenic *E. coli* causing human disease (Rodriguez-Siek et al., 2005; Vincent et al., 2010; Manges and Johnson, 2012; Manges, 2016). In a study comparing the incidences of antimicrobial resistance across food products (such as vegetable salads and raw meats), raw chicken was reported to have the highest *E. coli* incidence (23.3%) of antimicrobial resistance (Rasheed et al., 2014). Food products contaminated with *E. coli* continue to be a food safety concern and cause of economic losses to the poultry industry, especially as production converts to antibiotic-free programs (Fancher et al., 2020).

*E. coli* is a Gram-negative bacteria, commonly found in intestinal tracts of animals and humans, that can cause a variety of infections and contribute to the spread of antimicrobial resistance (Rasheed et al., 2014). Chickens can serve as hosts for antimicrobial-resistant *E. coli* because there are multiple routes of contamination at each stage of poultry production. Transmission of antimicrobial resistant genes can be attributed to (plasmid-mediated) beta-lactamases, efflux pumps, aminoglycoside phosphorylases, hydrolases, and chloramphenicol transacetylase, among others (Dame-Korevaar et al., 2019; Pandey and Cascella, 2022). High levels of antimicrobial resistance have been found in day-of-hatch chicks (Braykov et al., 2016), which could originate from the birds’ intestinal microbiota (from vertical transmission) and from the hatchery environment itself (Osman et al., 2018). Bacterial horizontal transmission occurs within and between flocks, which leads to widespread transmission from the farm to the environment (Dame-Korevaar et al., 2019). Regardless of the origin, *E. coli* is capable of developing resistance through the acquisition of resistance genes via mutations and horizontal gene transfer (Ievy et al., 2020).

A single bacterium can quickly transfer an antimicrobial-resistant gene and spread resistance to the rest of the bacterial colony (Martinez and Baquero, 2000). In the United States, broiler chicken litter is commonly reused on-farm for several flocks, so long as the litter is properly managed to reduce pathogenic bacteria (Stenutz et al., 2006). Previous studies have also indicated a high prevalence (63–100%) of multi-drug resistant *E. coli* (Kyakuwaire et al., 2019). While proper management of reused litter is not harmful and can be beneficial, the mismanagement of reused litter could be a source of not only pathogenic bacteria, but also antimicrobial resistance.

The objective of this study was to identify and characterize the antimicrobial resistance profile of *E. coli* isolated from litter collected from 16 pens that contained broiler chickens. Relationships between phenotypic resistance and virulence genes harbored by *E. coli* isolates known to contribute to the prevalence of antimicrobial resistant bacteria were investigated.

## MATERIALS AND METHODS

### Location

Litter samples were collected at the end of a concurrent broiler chicken experiment conducted from September to November 2020. The birds were not vaccinated or treated with any antimicrobials. All animal procedures were approved by the University of Maryland Institutional Care and Use Committee (protocol #R-JUL-20-35). Briefly, 400 day-of-hatch Ross 708 broiler chicks were placed into 16 pens within 2 rooms in the Animal Wing on campus at the University of Maryland. Within each room, 8 pens were placed with 25 birds per pen. Each pen was 1.5 m (5 feet) wide by 3 m (10 feet) long and contained new aspen wood shavings. The current study was conducted because the birds in a concurrent study became ill and on d 29, a subset of culled birds was confirmed to be infected with pathogenic *E. coli*, along with Infectious Bronchitis, *Enterococcus durans*, and *Enterococcus faecium*, by the Maryland Department of Agriculture Animal Health Diagnostic Laboratory in Frederick, Maryland. Litter samples were collected on d 53. Composite litter samples were collected from approximately 1 cup of litter from 15 locations within each pen (5 locations under the waterline near the back of the pen, 5 locations near the middle of the pen, and 5 locations at the front of the pen), homogenized by hand, and stored at −20°C until further analysis.

### Isolation of *E. coli*

In the laboratory, litter samples were thawed to room temperature and 3 subsamples from each of the 16 pens (n = 48 subsamples), ranging from 0.10 to 0.30 g, were aseptically placed into 15-mL conical tubes with 10 mL of tryptic soy broth (**TSB**; BD Difco, Sparks, MD**)**. Samples were incubated overnight at 37°C. Following incubation, 100 µL of the TSB solution was streaked onto MacConkey agar plates (BD Difco**)**. The plates were incubated overnight at 37°C. The next day, 3 individual bacterial colonies visually resembling *E. coli* were selected from each MacConkey agar plate, placed into TSB, incubated overnight at 37°C, and streaked onto tryptic soy agar (**TSA**; BD Difco) plates the next day. Of the 48 subsamples, a total of 68 *E. coli* isolates were found.

### Antimicrobial Susceptibility

Antimicrobial susceptibility of *E. coli* isolates was examined using the disc diffusion method, with *Escherichia coli* strain ATCC 25922 as the control. Guidelines set forth by the Clinical and Laboratory Standards Institute (CLSI, 2017) and Bauer, et al. (1966) were used for susceptibility classification. Briefly*,* isolates were stored in a suspension of Luria-Bertani (**LB**) broth (BD Difco) and 20% glycerol at −20°C. Isolates were streaked onto TSA, incubated overnight at 37°C, and individual colonies were selected, and inoculated into 5 mL of Milli-Q water until the bacterial suspension reached an optical density measured at 600 nm (OD 600) of 0.08 to 0.13 using a spectrophotometer (NanoPhotometer, Implen, München, Germany). The bacterial suspensions were vortexed and streaked onto a Mueller Hinton Agar plate (BD Difco). Each of the 68 isolates were tested against 10 antimicrobials: ampicillin (AMP; 10 µg), azithromycin (AZM; 15 µg), colistin (CT; 10 µg), imipenem (IPM; 10 µg), norfloxacin (NOR; 10 µg), streptomycin (STR; 10 µg), sulphonamides (S; 300 µg), trimethoprim/sulfamethoxazole (SXT; 25 µg), tetracycline (TE; 30 µg), cephalothin (KF; 30 µg) (Oxoid, Basingstoke, UK). After incubation at 37°C for 18 hours, resistance breakpoints were determined from the CLSI (2017), except colistin, for which the guidelines in Bauer et al., (1966) were used. For all antimicrobials, isolates were recorded as resistant to the antimicrobial if the zone diameter (mm) was at or below the CLSI recommended breakpoint. *E. coli* isolates were classified as either resistant if the zone diameter was less than the breakpoints, or susceptible if the zone diameter was greater than the breakpoints. To compare the binomial response of either resistant or susceptible, the isolates that exhibited intermediate susceptibility to the antimicrobials were also categorized as resistant.

### DNA Extraction

Bacterial DNA was obtained from the whole organisms using the boil prep method (Barbieri et al., 2013). Briefly, isolates were grown at 37°C overnight on LB agar (BD Difco). Next, an isolated colony was inoculated into 1 mL of LB broth and grown overnight at 37°C. Cultures were centrifuged at 12,000 RCF for 3 min, the supernatant was discarded, cells were re-suspended in 200 μL of molecular-grade water, and boiled at 100°C (Isotemp, Fisher Scientific, Dubuque, IA) for 10 min. After cooling, the suspension was centrifuged at 12,000 RCF for 3 min to precipitate cellular debris, and 150 μL of the supernatant was transferred to a new tube and used as the DNA template for gene amplification. The DNA extracts were stored at −20°C until use.

### Polymerase Chain Reaction Amplification

Polymerase chain reaction (**PCR**) analysis for O-antigen serotyping (Iguchi et al., 2015), characterizing antimicrobial resistance and heavy metal genes (Johnson et al., 2008b), and APEC minimal predictor genes (Johnson et al., 2008a) was carried out using the following protocol with minor modifications for annealing temperatures of the primers (de Oliveira et al., 2020; Barbieri et al., 2021; Newman et al., 2021).

All PCR reactions were prepared in a total volume of 25 µL for each isolate. Components for the PCR reaction consisted of 2.5 µL of 10X PCR buffer, 0.4 µL of 10 mM MgCL_2_, 1 µL of 0.2M dNTP mixture, 2 µL of TAQ polymerase (Dream TAQ, ThermoFisher, Waltham, MA), 1.2 µL of primer pool, 2 µL of DNA, and 15.9 µL of sterile molecular grade water. Positive control strains were included in the analysis for the appropriate genes of interest from previously characterized strains in our lab collection (Johnson et al., 2008a; de Oliveira et al., 2020; Barbieri et al., 2021; Newman et al., 2021), and the negative controls included sterile water in place of DNA. Amplification parameters of the thermocycler (Mastercycler X50, Eppendorf, Hamburg, Germany) included an initial denaturing step at 94°C for 5 min, followed by 30 rounds of 94°C for 30 s, 63°C for 30 s, 68°C for 3 min, then a final extension of 72°C for 10 min, and a hold at 4°C.

The generated PCR products were subjected to electrophoresis, performed in a 2% agarose gel (Agarose LE, Lonza, Alpharetta, GA) running at 100 V for 90 min. The gel was stained with ethidium bromide (0.25%) solution for 20 min, visualized using an imager (UVP BioDock-It^2^ Imager, Analytik Jena, Jena, Germany), and analyzed for the presence of PCR products of the appropriate size when compared with the lab control strains for the targeted genes.

### 16s *E. coli* Confirmation

Isolates of typical morphology from the MacConkey agar plates were identified as *E. coli* and confirmed with a 16S rRNA PCR that was performed for each sample (Lamprecht et al., 2014). Amplification of the gene target was carried out as described above.

### Antimicrobial and Heavy Metal Resistance Genes

DNA samples were amplified using PCR in multiplex panels to amplify a series of common antimicrobial resistance genes and heavy metal resistance genes harbored by *Enterobacteriaceae* species. The genes of interest were: *blaTEM, aac 3VI, tetB, tetA, groEL. aph(3)IA, dfr17, silP, intl1, pcoD, sull, ISEc12, aadA, aac3-VI,* and *qacEdelta1* (Johnson et al., 2008b; de Oliveira et al., 2020; Barbieri et al., 2021; Newman et al., 2021).

### Serotyping

The *E. coli* isolates were tested for their O-antigen serogroup using PCR (Iguchi et al., 2015). The O-antigen serogroups included in the PCR were O1, O2, O8, O15, O18, O25, O26, O29, O30, O55, O75, O78, O84, O86, O8,8 O91, O103, O111, O113, O115, O119, O121, O12,8 O13,2 O138, O145, O150, O152, O157, O160, O161, O165, O166, and O183.

### APEC-Like Minimal Predictors

A multiplex PCR was performed with the confirmed *E. coli* isolates to identify the presence or absence of genes found in APEC isolates (Johnson et al., 2008a,2008b; Logue et al., 2012). The detection of at least 3 of the 9 plasmid (*cvaC, iroN, ompTp, hlyF, etsB, iss, aerJ*), chromosomal (*ireA, papC*), or a combination of the plasmid and chromosomal genes classified the *E. coli* isolates as APEC-like. The genes used to classify APEC-like strains cannot definitively conclude that the isolated *E. coli* are truly APEC because they were isolated from the litter and not directly isolated from diseased birds*,* but they are likely prospects (Johnson et al., 2008a). DNA samples were amplified using PCR in multiplex panels to amplify a series of 23 virulence-associated genes in APEC.

### Statistical Analysis

To determine if the presence of the virulence genes were more likely to be present in isolates that were resistant to antimicrobials, a chi-square contingency analysis for each pair of antimicrobials and genes was performed in JMP Pro (version 14.2, SAS Institute, Inc., Cary, NC). Data was considered significant at *P* ≤ 0.05.

## RESULTS

### Antimicrobial Susceptibility

**Figure 1:**
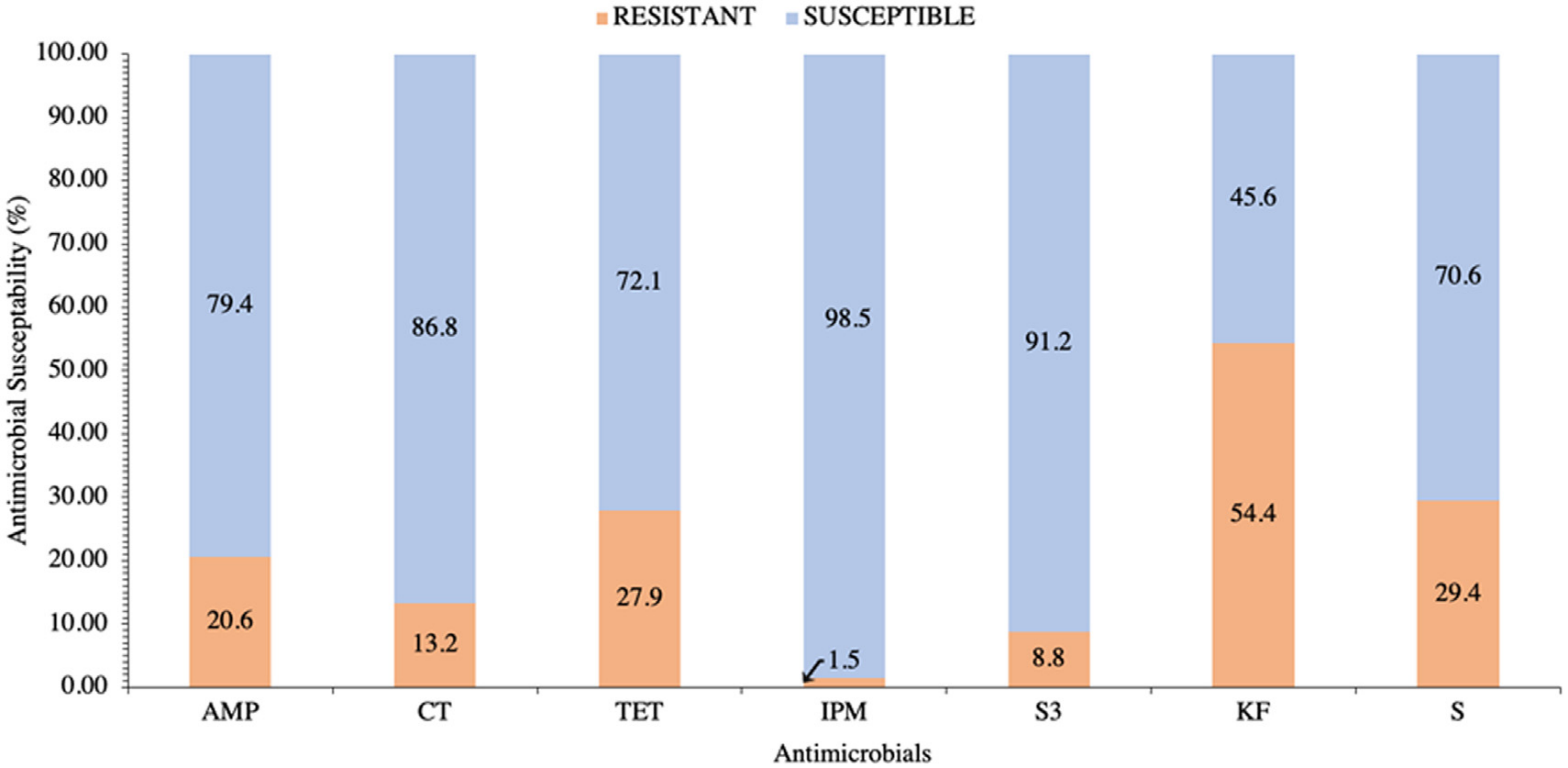
Antimicrobial susceptibility prevalence of *E. coli* isolates (n = 68) from the litter of broiler chickens raised in 16 pens and tested for the phenotypic resistance (orange) or susceptibility (blue) to ampicillin (AMP), colistin (CT), tetracycline (TET), imipenem (IPM), sulphonamides (S3), cephalothin (KF), and streptomycin (S). All isolates were susceptible to azithromycin, sulfamethoxazole/trimethoprim, and norfloxacin and are not shown.

The zones of inhibition of the 68 isolates to 10 antimicrobial discs in the disc diffusion assays were measured to determine antimicrobial susceptibility/resistance. The abbreviations used for each antimicrobial in Tables 1 and 2 and Figure 1 are as follows: ampicillin (AMP), colistin (CT), tetracycline (TET), sulphonamides (S3), cephalothin (KF), and streptomycin (S).

**Table 1:** Counts of *E. coli* isolates (n = 68) sourced from the litter of broiler chickens raised in 16 pens that were susceptible to all antibiotics and unique patterns of resistance to cephalothin (KF), streptomycin (S), tetracycline (TET), ampicillin (AMP), colistin (CT), sulphonamides (S3), and imipenem (IPM).

Antimicrobial						# Isolates
AMP						1
KF						11
S						2
TET						2
AMP	KF					2
KF	S					4
CT	KF					3
CT	TET					1
TET	KF					2
TET	S					3
AMP	KF	S				1
AMP	CT	KF				1
CT	TET	KF				2
TET	KF	S				2
AMP	S3	KF	S			1
AMP	TET	KF	S			2
AMP	TET	S3	KF			1
AMP	CT	S3	KF	S		1
AMP	CT	TET	KF	S		1
AMP	TET	S3	KF	S		2
AMP	TET	IPM	S3	KF	S	1
Total Susceptible						22
Total						68

**Table 2:** The cumulative prevalence and individual isolate presence (black) or absence (white) of antimicrobial resistance O-type gene expression, antimicrobial and heavy metal resistance genes, and genes indicative of avian pathogenic *E. coli* (APEC) of *E. coli* isolates (n = 68) from the litter of broiler chickens raised in 16 pens. Antimicrobials are abbreviated as AMP (ampicillin), CT (colistin), TET (tetracycline), IMP (imipenem), S3 (sulphonamides), KF (cephalothin), and S (streptomycin) and genes are *tetB, tetA, groEL, aph(3)IA, silP, intl1, pcoD, sull, ISEc12, aadA, aac3-VI, qacEdelta1, cvaC, iroN, ompTp, hlyF, etsB, iss, aerJ,* and *ireA*.

All isolates were susceptible to azithromycin, trimethoprim/sulfamethoxazole, and norfloxacin, and the cumulative resistance of isolates to the other 7 antimicrobials is summarized in Figure 1. Of the isolates that exhibited resistance, the lowest percentage was to imipenem (1.5%) and the greatest was to cephalothin (54.4%) (Figure 1). The percentage of isolates that exhibited resistance to sulphonamides, colistin, ampicillin, tetracycline, and streptomycin were 8.8%, 13.2%, 20.6%, 27.9%, and 29.4%, respectively.

Phenotypic multidrug resistance profiles are presented in Table 1. A total of 22 (32.4%) isolates were susceptible to all 10 antimicrobials (Table 1). The remaining 46 (67.6%) isolates exhibited 21 unique patterns of antimicrobial resistance. The most prevalent profiles of isolate resistance were to: cephalothin (16.2%); cephalothin and streptomycin (5.9%); colistin and cephalothin (4.4%); and tetracycline and streptomycin (4.4%). Multidrug resistance, which is resistance to 3 or more antimicrobials, was found in 22.1% of the isolates (Table 1). Resistance to 1 antimicrobial was found in 23.5% of the isolates; 22.1% were resistant to 2 antimicrobials; 8.8% were resistant to 3 antimicrobials; 5.9% were resistant to 4 antimicrobials; 5.9% were resistant to 5 antimicrobials, and 1.5% of the isolates were resistant to 6 antimicrobials (Table 1). Resistance/susceptibility profiles for each isolate are shown in Table 2.

### O-Antigen Serotyping

The PCR-based method was used to determine that a total of 4 O-serogroups were identified from 20.6% isolates and of those isolates 57.1% were the serotype O15, 21.4% were O91, 14.3% were O78, and 7.1% were serotype O75 (Table 2).

### PCR Amplification of Heavy Metal Genes and Antimicrobial Resistance Genes

**Figure 2:**
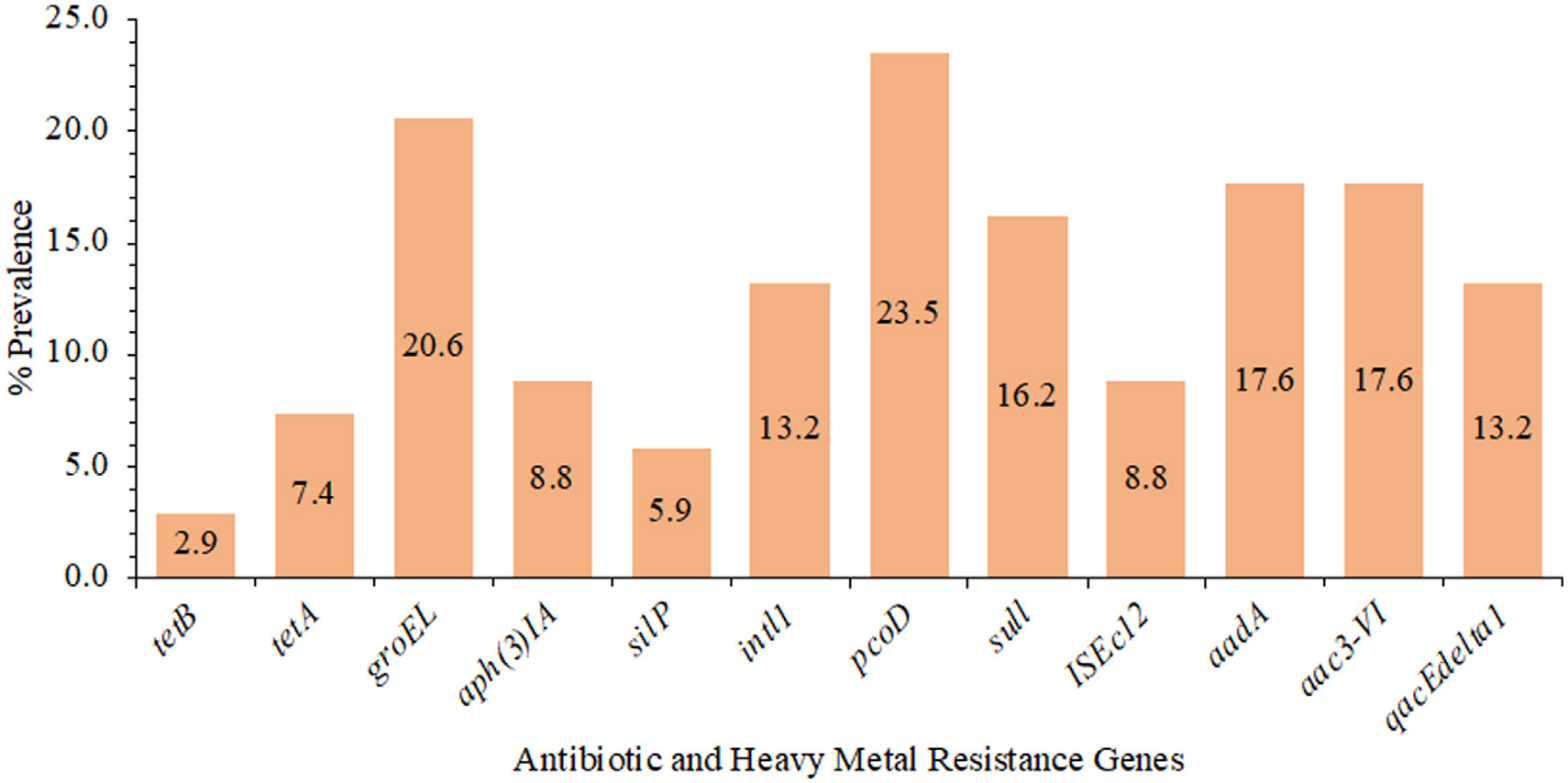
The prevalence (%) of antimicrobial (*blaTEM, aac 3VI, tetB, tetA, aph(3)IA, dfr17, aadA, aac3-VI*) and heavy metal (*silP, pcoD, sull, qacEdelta1*) resistance genes and essential functional genes (*groEL, intl1, ISEc12)* of *E. coli* isolates (n = 68) from the litter of broiler chickens raised in 16 pens. The *blaTEM, dfr17*, and *aac 3VI* gene were not present in any of the isolates.

Antimicrobial and heavy metal resistance gene prevalence are presented in Figure 2. The most prevalent genes were *pcoD* at 23.5% and *groEL* at 20.6%, and *tetB* was the least prevalent, found in 2.9% of the isolates. At least 1 antimicrobial or heavy metal resistance-associated gene was present in 69.1% of the isolates. One antimicrobial resistance gene, heavy metal resistance gene, or both were present in 35.3% of the isolates, 2 genes were detected in 13.2%, 3 genes in 10.3%, 4 genes in 0.0%, 5 genes in 2.9%, 6 genes in 2.9%, and 7 genes in 4.4% of isolates examined (Table 2).

### APEC-like Minimal Predictors

**Figure 3:**
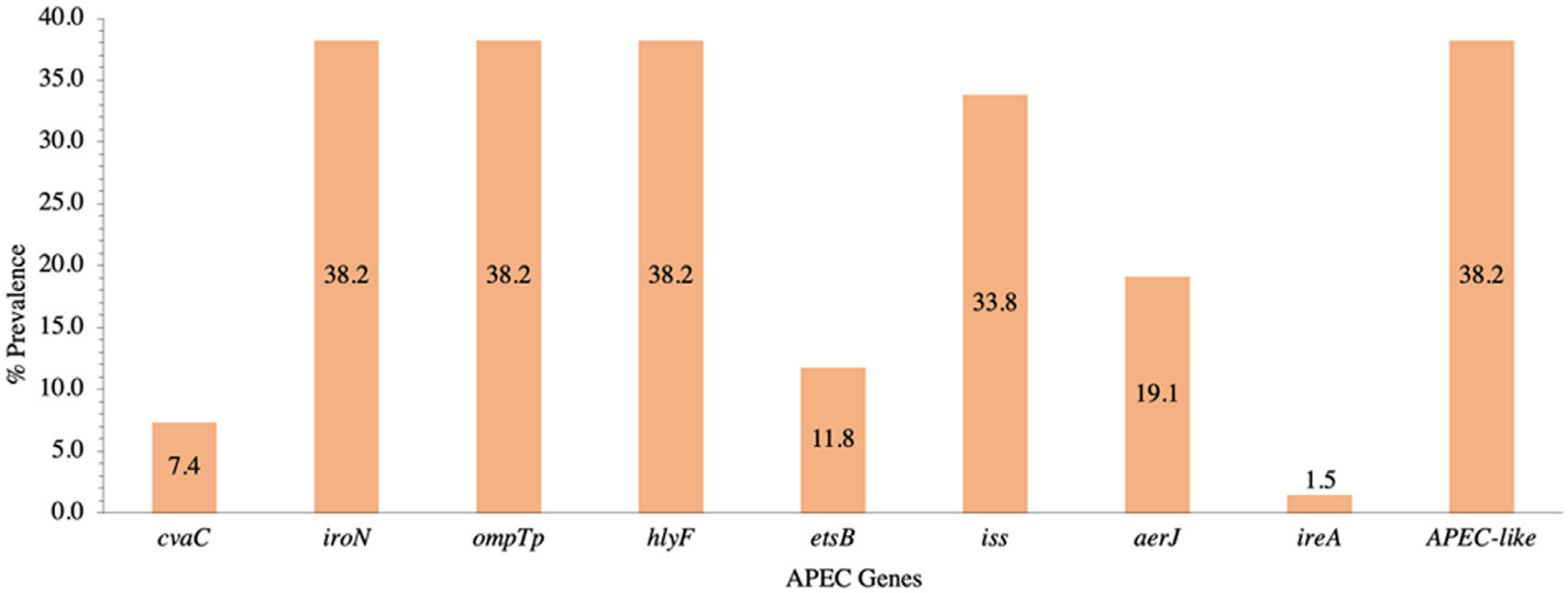
The prevalence (%) of 9 genes (*cvaC, iroN, ompTp, hlyF, etsB, iss, aerJ, ireA,* and *papC*) as minimal predictors of avian pathogenic *E. coli* (APEC) from isolates (n = 68) sourced from the litter of broiler chickens raised in raised in 16 pens. The presence of at least 3 genes is needed to be categorized as APEC-like. The *papC* gene was not present in any of the isolates.

A total of 9 APEC genes were assayed (*cvaC, etsB, aerJ, iss, iroN, ompTp, hlyF, ireA,* and *papC*), and isolates were considered APEC-like if they possessed 3 or more APEC genes (Johnson et al., 2008a). Of the 68 isolates, 38.2% were considered APEC-like (Figure 3). None of the isolates possessed the *papC* gene. The most prevalent genes were *iroN, ompTp*, and *hlyF*; each found together in 38.2% of isolates and occurred concurrently in 96.2% of those isolates (Figure 3). Isolates also had *iss, aerJ, etsB, cvaC,* and *ireA* at 33.8, 19.1, 11.8, 7.4, and 1.5%, respectively (Figure 3).

### Antimicrobial Phenotypic Resistance and Genotype

The phenotypic resistance of isolates and the genes that they presented were assessed for potential relationships. Of the isolates that were resistant to cephalothin (n = 37 isolates), 85.7% (12/14) were positive for *groEL* and 100.0% (5/5) were positive for *tetA* genes*.* The chi-square analysis showed a significant association between cephalothin resistance and the genes *groEL* (*P =* 0.0009) and *tetA (P* = 0.04*)*.

Of the isolates resistant to tetracycline (n = 19 isolates), 100.0% (5/5) harbored *tetA*, 57.1% (8/14) harbored *groEL,* 58.3% (7/12) harbored *aadA,* 58.3% (7/12) harbored *aac3-VI,* and 66.7% (6/9) habored *qacEdelta1.* The chi-square analysis showed a significant association between tetracycline resistance and the genes *tetA (P* = 0.001*), groEL (P* = 0.01*), aadA (P* = 0.02*), aac3-VI* (*P* = 0.02), and *qacEdelta1* (*P* = 0.01)*.*

Of the isolates resistant to streptomycin (n = 20 isolates), 56.3% (9/16) presented the *pcoD* gene, and 75.0% (9/12) presented the *aadA* gene. The chi-square analysis showed a significant association between streptomycin resistance and the genes *pcoD* (*P* = 0.01) and *aadA* (*P* = 0.0004).

Of the isolates resistant to ampicillin (n = 14 isolates), 42.9% (6/14) presented *groEL,* 66.7% (4/6) presented *aph(3)IA,* 75.0% (3/4) presented *silP,* 37.5% (6/16) presented *pcoD,* 72.7% (8/11) presented *sull,* 58.3% presented (7/12) *aadA,* 66.7% (6/9) presented *qacEdelta1,* 34.6% (9/26) presented *iroN,* 34.6% (9/26) presented *ompTp,* and 34.6% (9/26) presented *hlyF.* The chi-square analysis showed a significant association between ampicillin resistance and the genes *groEL* (*P* = 0.02)*, aph(3)IA* (*P* = 0.01), *silP* (*P* = 0.02)*, sull* (*P* = 0.0001), *aadA* (*P* = 0.002)*, qacEdelta1 (P* = 0.002*), iroN* (*P* = 0.03)*, ompT (P* = 0.03)*,* and *hlyF (P* = 0.03*).*

Of the isolates resistant to colistin (n = 9 isolates), 35.7% (5/14) presented the *groEL* gene*.* The chi-square analysis showed a significant association between colistin resistance and the *groEL* gene (*P* = 0.01).

Of the isolates that exhibited resistance to sulphonamides (n = 6), 60.0% (3/5) presented for *tetA,* 35.7% (5/14) presented *groEL*, 66.7% (4/6) presented *aph(3)IA*, 75.0% (3/4) presented *silP*, 25.0% (4/16) presented *pcoD*, 54.5% (6/11) presented *sull*, 50.0% (6/12) presented *aadA*, and 66.7% (6/9) presented *qacEdelta1* gene. The chi-square analysis showed a significant association between sulphonamide resistance and the genes *tetA* (*P* = 0.004), *groEL* (*P* = 0.001), *aph(3)IA* (*P* = 0.0003), *silP* (*P* = 0.002), *pcoD* (*P* = 0.02), *sull* (*P* = 0.0001), *aadA* (*P* = 0.0001), and *qacEdelta1* (*P* = 0.0001).

There were no significant associations for genes with imipenem resistance. Isolates that were resistant to cephalothin, tetracycline, and streptomycin did not have significant associations with APEC genes. However, there was a greater likelihood that APEC genes *iroN, ompTp,* and *hlyF* were present when isolates were resistant to ampicillin (*P* = 0.03).

## DISCUSSION

The present study investigated *E. coli* isolated from the litter of broiler chickens to characterize antimicrobial susceptibility and to determine the relationship between phenotypic resistance and select virulence genes.

Antimicrobial resistance in the poultry industry is of great concern. *E. coli* can have significant impacts on consumers when purchasing poultry meat, and on physicians and patients such as in the treatment of urinary tract infections (Barbieri et al., 2017). Of the 68 isolates sampled from the litter of broilers in our study, the greatest phenotypic resistance was to cephalothin (54.4%). These results align with those of previous studies, which have also indicated high prevalence of *E. coli* resistance to cephalothin in poultry. In another study, cloacal swabs from birds with a history of colibacillosis were used in the analysis of cephalothin resistance of 30 *E. coli* isolates from broiler farms in Thailand, with a resistance rate of 73% (Mooljuntee et al., 2010). Similarly, a study originating from Korea isolated 591 *E. coli* isolates from both feces and dust and reported that the first generation cephalosporins had the highest incidences of resistance, ranging from 60 to 71% (Seo et al., 2019).

Tetracycline has been registered for use in the United States, China, Poland, United Kingdom, France, Brazil, and Spain for therapeutic, metaphylactic/prophylactic, and growth promotion purposes for over 50 yr (Barbieri et al., 2015; Roth et al., 2019). Resistance to tetracycline is associated with large plasmids that encode efflux genes which regulate the internal environment of the Gram-negative bacteria (Soto, 2013). In *E. coli,* these large plasmids can also carry other genes such as those responsible for pathogenic factors, antimicrobial resistance, and heavy metal resistance (Diarrassouba et al., 2007). A study that analyzed the susceptibility of 144 APEC isolates from cellulitis lesions of broiler chickens found that 69% of isolates exhibited resistance to tetracycline (Barbieri et al., 2013), while our study indicated that 27.9% of *E. coli* isolates were resistance towards tetracycline. A study by Smith et al. (2007) analyzed cecal droppings found on the surface of the litter at 3 untreated commercial broiler farms at wk 3 and 6. Their study sampled 450 *E. coli* isolates and indicated a greater prevalence of tetracycline resistance, ranging from 36% at wk 3 to 97% at wk 6 (Smith et al., 2007). Previous work with *E. coli* in Brazil investigated 52 APEC isolates from systemic colibacillosis and observed that 69% of isolates exhibited tetracycline resistance (Barbieri et al., 2015). Our study also indicated moderate resistance to tetracycline, likely attributed to its long and common use in poultry, which is supported by the finding of tetracycline resistant bacteria in birds that were not administered this antimicrobial (van den Bogaard and Stobberingh 2000; Agyare, 2019). In general, the lower rates of tetracycline resistance in the current study compared to the previous studies could be a result of the absence of tetracycline and antimicrobial administration. Further, the use of tetracycline in the poultry industry has greatly reduced since 2015 after U.S. FDA implementation of the GFI #209 (Singer et al., 2020). Consequently, less antimicrobial residues would have been shed from the broilers and less instances of *E. coli* resistance to tetracycline would be observed.

Streptomycin is approved for use in Brazil and is seldom used in the United States for the same purposes as listed for tetracycline (Roth et al., 2019). In our study, 27.9% of *E. coli* isolates exhibited resistance to tetracycline, which was lower compared to other work that also isolated *E. coli* from diseased broilers that were not treated with any antimicrobials. According to 2 different studies in Egypt, there were greater instances of streptomycin resistance reported at 74% (Younis et al., 2017) and 80% resistance (Amer et al., 2018). Smith et al. (2007) provided a range of 53 to 100% of *E. coli* streptomycin resistance from 3 untreated commercial broiler houses for the United States. In Nigeria, Okorafor et al. (2019) observed a range of 10 to 80% of streptomycin resistance in broiler chicks from 4 different hatcheries. The results from our study are lower than the findings of these previous studies, which may be attributed to the single (litter) source of our *E. coli* isolates or that birds were reared in a controlled research setting in the current study.

The prevalence of *E. coli* ampicillin resistance in this study was 20.6% and this is also lower than findings of previous studies. A study originating from Thailand collected 30 cloacal swabs from broilers on commercial farms and found that 100% of the *E. coli* isolates exhibited ampicillin resistance (Mooljuntee et al., 2010). From the studies mentioned previously by Younis et al. (2017) and Amer et al. (2018), 47 and 80% of *E. coli* isolates exhibited ampicillin resistance, respectively. In Nigeria, Okorafor et al. (2019) observed that 80 to 100% of the isolates from broiler chicks exhibited ampicillin resistance. Ampicillin is approved for use in Brazil, China, Germany, and France (Roth et al., 2019), but is not approved for use in the United States for any purpose in poultry and livestock, which may explain our study's lower prevalence of ampicillin resistance.

Interestingly, 13.2% of *E. coli* isolates were resistant to colistin in our study. Resistance to colistin is concerning because it is a last-resort antimicrobial used to treat bacterial infections in humans. The plasmid-mediated mobile colistin resistance (*mcr*) gene encodes colistin resistance (Barbieri et al., 2017), and it is mostly found in *E. coli* isolated from swine, bovine, poultry, and their related products (Valiakos and Kapna, 2021). The use of colistin in poultry was approved in many countries by their respective national regulatory authorities, including the United States, Brazil, China, Poland, the United Kingdom, France, Spain, and Germany (Roth et al., 2019) and were banned between 2015 and 2016 in Brazil Japan, India, and China (Liu et al., 2016; Schoenmakers, 2020). While colistin is approved for use in the United States, it is not approved for commercial poultry use. The potential benefits of this ban are significant in reducing the use of antimicrobials and decreasing the spread of antimicrobial resistance genes; however, it does not offer a direct solution to the present public health concerns caused by antimicrobial resistant bacteria and their genes.

Our study indicated minimal (1.5%) resistance to imipenem, and none of the isolates presented a significant association with any of the genes of interest. Imipenem resistance is mostly associated with the Gram-negative Enterobacteriaceae *Klebsiella pneumoniae (K. pneumoniae)* that produces *K. pneumoniae* carbapenemase (Karlsson et al., 2022). A study by Karlsson et al. (2022) surveyed carbapenemase producing and non-carbapenemase producing bacteria collected from clinical laboratories in 8 U.S. states. Of the 419 isolates analyzed, only 23 *E. coli* isolates exhibited carbapenem resistance compared to the 242 *K. pneumoniae*-resistant isolates (Karlsson et al., 2022). It could be that the minimal imipenem resistance seen in our study is due to the mechanism of carbapenem resistance, which depends on mobile genetic elements derived from *K. pneumoniae* (Nordmann and Poirel, 2019)*.* Since the birds in our study were confirmed to be infected with *E. coli* and not *K. pneumoniae,* a future endeavor would include surveying the microbial population of the broiler litter in addition to testing for antimicrobial resistance.

Sulphonamide resistance was exhibited in 8.8% of our isolates. This prevalence of resistance is in the lower range of our study. Sulphonamide use in the poultry industry is limited due to the high potential for the birds to develop toxic side effects (Landoni and Albarellos, 2015). A previous study by Furtula et al. (2010) assessed the antimicrobial resistance of *E. coli* in both commercial and controlled broiler feeding trials. It was suggested that *E. coli* resistance to sulphonamides was present in the chicks since hatch because of the existing sulphonamide resistant *E. coli* found in the birds of the control group at d 36 (Furtula et al., 2010). Since the isolates resistant to sulphonamides originated from birds that were housed in pens in the same facility, it is possible that as chicks, the birds used in our study were from a breeder flock where sulphonamide resistance existed.

This study also sought to identify genes indicative of avian pathogenic *E. coli.* As described by Johnson et al. (2008a), there are genes that can be indicative of APEC, and isolates must have at least 3 of the following 9 genes to be considered APEC-like: *cvaC, iroN, ompTp, hlyF, etsB, iss, aerJ, ireA,* or *papC.* As a plasmid of interest, ColV is thought to assist APEC strains in infection (Barbieri et al., 2013). The ColV plasmid is associated with genes such as *cvi/cva, iroN, iss, iucD, sitD, traT,* and *tsh* (Barbieri et al., 2015). Our study found that the genes *iroN, ompTp,* and *hlyF* were the most prevalent (38.2%) among isolates and that all 3 genes were present together 96.2% of the time. Although our study did not assess plasmid genes as in previous studies (Peigne et al., 2009; Mahjoub-Messai et al., 2011), it is possible that the isolates that contained the genes *iroN, ompTp, hlyF,* and *iss* could have shared the same virulence plasmid.

The isolates used for antimicrobial susceptibility testing were also assessed for specific genes relating to heavy metal and antimicrobial resistance. Our study found the greatest presence of the *pcoD* gene, followed by *groEL,* then *aadA* and *aac3-VI*. The *pcoD* gene is related to copper resistance, *groEl* encodes a chaperone protein, *aadA* is associated with streptomycin resistance, and *aac3-VI* is associated with gentamycin resistance (de Oliveira et al., 2020). A previous study isolated *E. coli* from the clinical material at St. Bartholomew's Hospital and indicated a linear proportional relationship in the resistance patterns of select aminoglycoside antimicrobials (Davies, 1971; Houang and Greenwood, 1977). This relationship indicates a cross resistance between streptomycin, neomycin, tobramycin, and kanamycin; however, previous studies found that *E. coli* isolates only showed a proportional increase in resistance between tobramycin and gentamycin (Davies, 1971; Houang and Greenwood, 1977). Similarly, our study found that streptomycin had one of the highest rates of resistance and there was a high prevalence of the *aadA* gene. It is possible that the same prevalence of the genes *aadA* and *aac3-VI* indicate that there could be cross-resistance to streptomycin and gentamycin.

## CONCLUSIONS

In summary, the *E. coli* isolates taken from litter used by broiler chickens in an experimental setting expressed phenotypic resistance to a wide range of antimicrobials from different classes as well as genes contiguous to their virulence. Regarding antimicrobial susceptibility, our isolates expressed moderate rates of resistance with the majority of isolates resistant towards cepthalothin. Virulence genes such as *iroN, ompT,* and *hlyF* were most prevalent in our isolates and frequently occurred simultaneously with one another, suggesting the presence of a conserved virulence plasmidic region. The relevancy of zoonoses, especially now, warrants greater efforts to investigate the intersection of agriculture and clinical medicine to not only reduce economic losses for farmers and producers but to also advance the breadth and depth of the One Health objective.
